# Supplementary material for: A theoretical foundation for state-transition cohort models in health decision analysis
Source: PLoS One. 2018 Dec 11;13(12):e0205543. doi: 10.1371/journal.pone.0205543 (PMC6289421; doi:10.1371/journal.pone.0205543)
Supplement: S1 Appendix — (PDF) [file pone.0205543.s001.pdf]

S1 Appendix. Mathematical derivations

Transition probability. In this section, we show the derivation for the probability of transitioning from S_k to state S_l in time step τ, which is given by:

$$P\left[S_k \to S_l \in (t, t+\tau] | n_k, t\right] = \nu_{kl}(\mathbf{n} - s_{kl}, t) = n_k c_{kl} \tau + o(\tau) \tag{1}$$

The probability of a *particular* transition $k \to l$ (R_{kl}) in a small time step τ is postulated to be linear in time: $c_{kl}\tau + o(\tau)$. This postulate is conceptually a first-order Taylor approximation to an instantaneous transition probability. The transition rate constant, c_{kl}, is interpreted as the derivative of the transition probability at time $\tau = 0$. We also restrict the number of allowable transitions in τ to one transition. If the number of individuals in S_k at time t is equal to n_k, then any of these n_k, assumed to be *indistinguishable*, individuals is at risk of transitioning. Since individuals are indistinguishable, each individual transition can be considered as a Bernoulli trial with a probability of success of $c_{kl}\tau + o(\tau)$. The probability of only one individual transitioning is equal to: $(c_{kl}\tau + o(\tau))(1 - c_{kl}\tau + o(\tau))^{n_k - 1}$. Since there are n_k ways of this transition to occur, we have:

$$P[S_k \to S_l \in (t, t+\tau] | n_k, t] = \binom{n_k}{1} (c_{kl}\tau + o(\tau))(1 - (c_{kl}\tau + o(\tau)))^{n_k - 1} \tag{2}$$

Equation $n_k c_{kl}(t)\tau + o(\tau)$ is then recovered as the τ terms of higher order are collected as $o(\tau)$ after expanding the binomial term in Eq 2.

Master equation. In principle, the master equation is based on the idea of mass conservation. First, the probability of observing a particular state-configuration at time $t + \tau$ is a function of the probabilities of the adjacent state-configurations at time t and the transitions between the corresponding probabilities of the state-configurations occurring in time step τ. The adjacent state configurations are defined as state-configurations with differences of $+1$ and -1 in two of the state counts, compared to the state-configuration of interest. The transitions between these corresponding probabilities are governed by the propensity function (ν). Mathematically, the first step translates to:

$$\begin{aligned} P(\mathbf{n}, t+\tau) &= \sum_{k=1}^{s} \sum_{l=1, l\neq k}^{s} \nu_{kl}(\mathbf{n} - s_{kl}, t) P(\mathbf{n} - s_{kl}, t) + \sum_{k=1}^{s} \sum_{l=1, l\neq k}^{s} (1 - \nu_{kl}(\mathbf{n}, t)) P(\mathbf{n}, t) \\ &= \sum_{k=1}^{s} \sum_{l=1, l\neq k}^{s} c_{kl}(t)(n_k + 1)\tau P(\mathbf{n} - s_{kl}, t) + \sum_{k=1}^{s} \sum_{l=1, l\neq k}^{s} (1 - c_{kl}(t) n_k \tau) P(\mathbf{n}, t) \end{aligned} \tag{3}$$

Rearranging Eq 3 and dividing it by τ yields:

$$\begin{aligned} \frac{P(\mathbf{n}, t+\tau) - P(\mathbf{n}, t)}{\tau} &= \sum_{k=1}^{s} \sum_{l=1, l\neq k}^{s} c_{kl}(t)(n_k + 1) P(\mathbf{n} - s_{kl}, t) \\ &- \sum_{k=1}^{s} \sum_{l=1, l\neq k}^{s} c_{kl}(t) n_k P(\mathbf{n}, t) \end{aligned} \tag{4}$$

The master equation is then established after taking the limit $\tau \to 0$.

Generating function method. A probability generating function (PGF) for a vector $\mathbf{x} = (x_1\ x_2\ \dots\ x_s)$ is defined by:

$$G(\mathbf{x},t) = \sum_{\mathbf{n}} x_1^{n_1} x_2^{n_2} \dots x_s^{n_s} P(\mathbf{n},t) \tag{5}$$

where $\sum_{\mathbf{n}} = \sum_{n_1} \dots \sum_{n_s}$. Differentiating $G(\mathbf{x},t)$ with respect to t and assuming that the series is uniformly convergent, we obtain:

$$\frac{\partial}{\partial t} G(\mathbf{x},t) = \sum_{\mathbf{n}} x_1^{n_1} x_2^{n_2} \dots x_s^{n_s} \frac{\partial}{\partial t} P(\mathbf{n},t)$$

or

$$\frac{\partial}{\partial t} G(\mathbf{x},t) = \sum_{\mathbf{n}} x_1^{n_1} x_2^{n_2} \dots x_s^{n_s} \sum_{k=1}^{s} \sum_{l=1}^{s} (\nu_{kl}(\mathbf{n} - s_{kl}, t) P(\mathbf{n} - s_{kl}, t) - \nu_{kl}(\mathbf{n},t) P(\mathbf{n},t) \tag{6}$$

Eq 6 can be simplified by (1) recognizing the following identity (e.g., for x_1):

$$\sum_{\mathbf{n}} n_1 x_1^{n_1 - 1} x_2^{n_2} \dots x_s^{s} P(\mathbf{n},t) = x_1 \frac{\partial}{\partial x_1} G(\mathbf{n},t),$$

(2) using the definition of PGF (Eq 5), and (3) rearranging the summation index to obtain the following first-order linear partial differential equation (PDE):

$$\frac{\partial}{\partial t} G(\mathbf{x},t) = \sum_{k=1}^{s} \sum_{l=1,,l \neq k}^{s} c_{kl}(x_l - x_k) \frac{\partial}{\partial x_k} G(\mathbf{x},t), \tag{7}$$

with an initial condition: $G(\mathbf{x},0) = \sum_{\mathbf{n}} x_1^{n_1} x_2^{n_2} \dots x_s^{n_s} P(\mathbf{n},0)$ The PDE by solved by using the method of characteristics. The characteristics equations are: $\frac{d\mathbf{x}(\xi)}{d\xi} = \mathbf{v}$ where $\mathbf{v} = (v_1 v_2 \dots v_s)$ and $v_i = \sum_{l=1, l \neq i}^{s} c_{ik}(x_l - x_i)$. Putting $\beta(s) = G(x(\xi), t - \xi)$, we have:

$$\frac{d\beta(\xi)}{d\xi} = \mathbf{v} \cdot \nabla G(\mathbf{x}(\xi), t - \xi) - \frac{\partial}{\partial t} G(\mathbf{x}(\xi), t - s\xi) \tag{8}$$

from which a general solution can be deduced: $G(\mathbf{x}(0), t) = G(\mathbf{x}(t), 0) = g(\mathbf{x}(t))$. The system of the characteristics equations can be written as:

$$\frac{d\mathbf{x}}{d\xi} = A\mathbf{x} \tag{9}$$

where A is an s x s, $(c_{kl})_{k,l \in \{1,2,\dots,s\}}$, matrix with elements of the form:
$A_{kl} = c_{kl} - \gamma_k \delta_{kl}$ with $\gamma_k = \sum_{l=1}^{s} c_{kl}$ and δ_{kl} is the usual Kronecker delta. Therefore, the solution of 9 takes the form:

$$\mathbf{x}(t) = B\mathbf{x}(0) \tag{10}$$

where $B(t) = \exp At = \sum_{k=1}^{\infty} \frac{(At)^k}{k!}$, i.e. the matrix exponential. Given, $G(\mathbf{x},0)$, $G(\mathbf{x}(0),t) = g(B\mathbf{x}(0))$. The solution of the PGF is then:

$$G(\mathbf{x},t) = \sum_{\mathbf{n}} \prod_{i=1}^{s} \left(\sum_{k=1}^{s} B_{ik}(t) x_k \right)^{n_i} P(\mathbf{n},0) \tag{11}$$

If we assumed all n_0 individuals start in state S_1, i.e. $x_1^{n_0}$, the solution of the PGF is then:

$$G(\mathbf{x},t) = \left(\sum_{k=1}^{s} B_{ik}(t)x_k\right)^{n_0} \tag{12}$$

The probability density function of the state-configuration can be recovered from Eq 12 by using the definition of the PGF. We introduce a vector: $\mathbf{z}_i = [z_{i1}\ z_{i2}\ \ldots\ z_{is}]$ where $i \in \{1,2,\ldots,s\}$ and the norm $||\mathbf{z}_i|| = z_{i1} + z_{i2} + \ldots + z_{is}$. Using the multinomial theorem:

$$(x_1 + x_2 + \ldots + x_s)^{n_i} = \sum_{||\mathbf{z}_i||=n_i} \frac{z_i!}{z_{i1}!z_{i2}!\ldots z_{is}!} x_1^{z_{i1}} x_2^{z_{i2}} \ldots x_s^{z_{is}},$$

we write

$$\left(\sum_{k=1}^{s} B_{ik}(t)x_k\right)^{n_i} = \sum_{\substack{\mathbf{z}_i \\ ||\mathbf{z}_i||=n_i}} n_i! \prod_{k=1}^{s} \frac{B_{ik}(t)}{z_{ik}!} x_k^{z_{ik}} \tag{13}$$

Therefore, we have:

$$\begin{aligned}
G(\mathbf{x},t) &= \sum_{\mathbf{n}} \prod_{i=1}^{s} \left(\sum_{k=1}^{s} B_{ik}(t)x_k\right)^{n_i} P(\mathbf{n},0) \\
&= \sum_{\mathbf{n}} \prod_{i=1}^{s} \left(\sum_{\substack{\mathbf{z}_i \\ ||\mathbf{z}_i||=n_i}} n_i! \prod_{k=1}^{s} \frac{B_{ik}(t)}{z_{ik}!} x_k^{z_{ik}} \right) P(\mathbf{n},0) \\
&= \sum_{\mathbf{n}} \sum_{\substack{\mathbf{z}_1\ldots\mathbf{z}_s \\ \|\mathbf{z}_1\|=n_1 \\ \vdots \\ \|\mathbf{z}_s\|=n_s}} \prod_{i=1}^{s} \left(\sum_{\substack{\mathbf{z}_i \\ ||\mathbf{z}_i||=n_i}} n_i! \prod_{k=1}^{s} \frac{B_{ik}(t)}{z_{ik}!} \right) \prod_{k=1}^{s} x_k^{\sum_{i=1}^{s} z_{ik}} P(n_1,n_2,\ldots,n_s,0) \\
&= \sum_{\mathbf{n}} \sum_{\substack{\mathbf{z}_1\ldots\mathbf{z}_s \\ \|\mathbf{z}_1\|=n_1 \\ \vdots \\ \|\mathbf{z}_s\|=n_s}} \prod_{i=1}^{s} \left(\sum_{\substack{\mathbf{z}_i \\ ||\mathbf{z}_i||=n_i}} \|\mathbf{z}_i\|! \prod_{k=1}^{s} \frac{B_{ik}(t)}{z_{ik}!} \right) \prod_{k=1}^{s} x_k^{\sum_{i=1}^{s} z_{ik}} P(\|\mathbf{z}_1\|,\|\mathbf{z}_2\|,\ldots,\|\mathbf{z}_s\|,0) \\
&= \sum_{\mathbf{n}} \sum_{\substack{\mathbf{z}_1\ldots\mathbf{z}_s \\ \mathbf{z}_1+\mathbf{z}_2+\ldots+\mathbf{z}_s=\mathbf{n}}} \prod_{i=1}^{s} \left(\|\mathbf{z}_i\|! \prod_{k=1}^{s} \frac{B_{ik}(t)}{z_{ik}!} x_k^{z_{ik}} \right) \prod_{k=1}^{s} x_k^{n_k} P(\|\mathbf{z}_1\|,\|\mathbf{z}_2\|,\ldots,\|\mathbf{z}_s\|,0)
\end{aligned}$$

If we rearrange the last line, we have:

$$\begin{aligned}
G(\mathbf{x},t) = \sum_{\mathbf{n}} & x_1^{n_1} x_2^{n_2} \ldots x_s^{n_s} \\
& \left[\sum_{\substack{\mathbf{z}_1\ldots\mathbf{z}_s \\ z_1+z_2+\ldots+z_s=\mathbf{n}}} \prod_{i=1}^{s} \left(\|\mathbf{z}_i\|! \prod_{k=1}^{s} \frac{B_{ik}(t)}{z_{ik}!} x_k^{z_{ik}} \right) P(\|\mathbf{z}_1\|,\|\mathbf{z}_2\|,\ldots,\|\mathbf{z}_s\|,0) \right]
\end{aligned} \tag{14}$$

Therefore the solution to the master equation is obtained after equating the coefficients in Eq 5 with Eq 11:

$$P(\mathbf{n},t) = \sum_{\substack{\mathbf{z}_1\ldots\mathbf{z}_s \\ z_1+z_2+\ldots+z_s=\mathbf{n}}} \prod_{i=1}^{s} \left(\|\mathbf{z}_i\|! \prod_{k=1}^{s} \frac{B_{ik}}{z_{ik}!} x_k^{z_{ik}} \right) P(\mathbf{n},0) \tag{15}$$

If we assumed all n_0 individuals start in state S_1, i.e. $x_1^{n_0}$, the solution takes the form of a multinomial distribution:

$$P(\mathbf{n}, t) = n_0! \prod_{m=1}^{s} \frac{B_{1m}^{n_m}}{n_m!} \tag{16}$$

Mean and variance of the master equation. The first moment of the master equation can be computed by differentiating Eq 12 and set all x equal to 1, e.g. the mean of the number of individuals in state S_i, given all n_0 individuals start in state S_1, is equal to:

$$\phi(t) = n_0 B_{1i}(t) \tag{17}$$

For an arbitrary initial distribution, the mean of the number of individuals in state S_i is given by:

$$\phi_i^a(t) = \sum_{\mathbf{n}} \sum_{k=1}^{s} n_k B_{ki}(t) P(\mathbf{n}, t) \tag{18}$$

The variance of the master equation can be derived by using the following relationship:

$$V_i(t) = \frac{\partial^2}{\partial {x_i}^2} G(\mathbf{x}, t) \bigg|_{\mathbf{x}=1} + \phi_i(t) - \phi_i(t)^2 \tag{19}$$

The variance of the number of individuals in state S_i, given all n_0 individuals start in state S_1, is equal to:

$$V_i(t) = n_0 B_{1i}(t)(1 - B_{1i}(t)) \tag{20}$$

The variance of the number of individuals in state S_i for an arbitrary initial distribution is given by:

$$V_i^a(t) = \sum_{\mathbf{n}} \left[\sum_{k=1}^{s} n_k B_{ki}(t) \left((n_k - 1) B_{ki}(t) + \sum_{l=1, l \neq k}^{s} n_l B_{li}(t) + 1 \right) \right] P(\mathbf{n}, t) + \phi_i^a(t)^2 \tag{21}$$
